# Developing a tablet computer-based application (‘App’) to measure self-reported alcohol consumption in Indigenous Australians

**DOI:** 10.1186/s12911-018-0583-0

**Published:** 2018-01-15

**Authors:** KS Kylie Lee, Scott Wilson, Jimmy Perry, Robin Room, Sarah Callinan, Robert Assan, Noel Hayman, Tanya Chikritzhs, Dennis Gray, Edward Wilkes, Peter Jack GradDipIndigH, Katherine M. Conigrave

**Affiliations:** 1 University of Sydney, Discipline of Addiction Medicine, Indigenous Health and Substance Use, NHMRC Centre of Research Excellence in Indigenous Health and Alcohol, King George V Building, 83-117 Missenden Road, Camperdown, NSW 2050 Australia; 2 Centre for Alcohol Policy Research, La Trobe University, 215 Franklin Street, Melbourne, VIC 3000 Australia; 3 Aboriginal Drug and Alcohol Council (ADAC) South Australia, 155 Holbrooks Road Underdale, Adelaide, South Australia 5032 Australia; 4 Alcohol, Tobacco and other Drugs Service, Queensland Health, 190 Palmerston Vincent, Townsville, QLD 4814 Australia; 5 Southern Queensland Centre of Excellence in Aboriginal and Torres Strait Islander Primary Health Care, 37 Wirraway Parade, Inala, QLD 4077 Australia; 6 School of Medicine, University of Queensland, Herston Road, Brisbane, QLD 4006 Australia; 7 School of Medicine, Griffith University, Gold Coast Campus, Gold Coast, Brisbane, QLD 4222 Australia; 8 National Drug Research Institute, Curtin University, 10 Selby St, Shenton Park, WA 6008 Australia; 9 Drug Health Services, Royal Prince Alfred Hospital, Sydney Local Health District, KGV Building, Missenden Road, Camperdown, NSW 2050 Australia

**Keywords:** Aboriginal, Indigenous, Alcohol, Measurement, Survey

## Abstract

**Background:**

The challenges of assessing alcohol consumption can be greater in Indigenous communities where there may be culturally distinct approaches to communication, sharing of drinking containers and episodic patterns of drinking. This paper discusses the processes used to develop a tablet computer-based application (‘App’) to collect a detailed assessment of drinking patterns in Indigenous Australians. The key features of the resulting App are described.

**Methods:**

An iterative consultation process was used (instead of one-off focus groups), with Indigenous cultural experts and clinical experts. Regular (weekly or more) advice was sought over a 12-month period from Indigenous community leaders and from a range of Indigenous and non-Indigenous health professionals and researchers.

**Results:**

The underpinning principles, selected survey items, and key technical features of the App are described. Features include culturally appropriate questioning style and gender-specific voice and images; community-recognised events used as reference points to ‘anchor’ time periods; ‘translation’ to colloquial English and (for audio) to traditional language; interactive visual approaches to estimate quantity of drinking; images of specific brands of alcohol, rather than abstract description of alcohol type (e.g. ‘spirits’); images of make-shift drinking containers; option to estimate consumption based on the individual’s share of what the group drank.

**Conclusions:**

With any survey platform, helping participants to accurately reflect on and report their drinking presents a challenge. The availability of interactive, tablet-based technologies enables potential bridging of differences in culture and lifestyle and enhanced reporting.

## Background

Data on the context in which people drink, what they drink, how much and how often, inform efforts to prevent and treat unhealthy alcohol use. While Aboriginal and Torres Strait Islander (Indigenous) Australians face up to eight times increased risk of harms from alcohol [1], there is a lack of good data on alcohol consumption itself [2, 3]. Some experts say that one national survey (published in 2008) underestimates consumption by more than 700% for females and 200% for males [4]. The national survey that is described as having the most suitable methods, and therefore most accurate data is more than two decades old and is specific to urban settings [2]. On a local level, communities and health services do not have a good way to monitor patterns of drinking and how well they are going with prevention or treatment efforts [5, 6].

Estimating how much alcohol an individual consumes is challenging in any population [7] and many approaches have been studied [2, 3, 8, 9]. None are perfect. Many methods require the drinker to convert their consumption into standard drinks or units. This requires awareness of the size of a standard drink (in Australia, equivalent to 10 g of ethanol), and then, awareness of the volume and strength, or standard drink content, of the beverage the person has consumed. The person then needs the mathematical skills to convert their consumption to standard drinks. Discomfort with reading or with numbers can be a significant [10, 11], which is more common in disadvantaged population subgroups. Estimating drinking by self-report is made more difficult if alcohol is shared, which can be common in the developing world and among indigenous peoples [11]. Episodic drinking patterns (e.g. due to geography, social or financial reasons or local alcohol restrictions) are also more common among Indigenous Australians, and make it difficult to answer questions on ‘usual’ drinking.

There is a need for a survey tool to collect comparable, standardised data on alcohol use, but which is flexible enough in terms of design and administration to be employed in, and responsive to, varying Indigenous contexts [3]. Alcohol tracker applications installed on a smartphone have been used to allow an individual to prospectively record their alcohol use [12]. However, this requires sustained participant engagement, and the availability of smartphones and internet, and so may not be feasible for large scale household surveys in indigenous or disadvantaged populations. Accordingly, a household survey tool which relies on retrospective reporting of drinking is likely to typically required. Compared to pen and paper surveys, or computer surveys which are purely text based, audio computer-assisted self-interviewing using tablet-computer technology or a similar platform may increase respondents’ engagement with survey items and increase their confidence in the anonymity and confidentiality of survey answers [13, 14]. Visual and audio opportunities offered by tablet-technologies may help counteract the need for individuals to be comfortable with numbers and the written word (in English; as is required with existing national alcohol paper and pen surveys). This paper discusses the processes used to develop a tablet computer-based application (‘App’) to collect a detailed assessment of drinking patterns in Indigenous Australians as a survey tool. The key features of the resulting App are described.

## Methods

### Overview

Study methods were designed by investigators in consultation with the Aboriginal Drug and Alcohol Council of South Australia (ADAC); the Aboriginal Drug and Alcohol Network New South Wales (ADAN), representing Aboriginal alcohol and other drug workers in New South Wales (NSW); and the Aboriginal Health Council of South Australia (AHCSA), the peak body for Aboriginal community controlled health services in South Australia (SA). Half the authors of this paper are themselves Aboriginal. Ethical approval was obtained from three ethics committees, including the the Aboriginal Health Council of South Australia (ACHSA) and Metro South Health Human Research Ethics Committee (Queensland).

The App was developed as part of a 5-year Australian National Health and Medical Research Council (NHMRC) project grant. That larger study aims to develop, test and re-test a tablet computer-based survey for Indigenous Australians (aged 16 years or older) to report on their drinking.

### Steps taken to consult with experts during App development

An iterative process was used during App development (instead of formal focus groups) with weekly or more frequent advice sought over a 12-month period from clinicians and other health professionals. This included from Indigenous alcohol and other drug, health, mental health, or health promotion workers; addiction medicine physicians; a nurse; a psychiatrist; Indigenous community leaders; and researchers of various expertise (including: epidemiology, sociology, survey design, psychometrics). Advice was also iteratively sought from other individuals from a range of backgrounds (see Table 1), by a smaller group of researchers (Lee, Conigrave, Wilson, Perry) and then relayed back to the App developers once consensus was reached. In particular, the Aboriginal Drug and Alcohol Council SA (ADAC; Wilson and Perry) played a lead advisory role in App development and in its deployment for validation.

**Table 1:** Grid showing skill area of individuals (*n* = 44) who advised on the development or testing of a tablet-based survey ‘App’ to help Indigenous Australians describe their drinking patterns

Skill area^a^	Indigenous (*n*)	Non-Indigenous (*n*)
Drug and alcohol (clinical)	7	4
Drug and alcohol (non-clinical)	11	–
Drug and alcohol (policy)	3	2
Mental health	1	1
Health promotion	2	1
Medicine	1	4
Psychology	–	2
Justice	2	–
Research (alcohol and other drugs)	5	11
Research (alcohol surveys, epidemiology, biostatistics, sociology, anthropology)	–	6
Proof reading	–	4
Community member	3	–
Total	**35**	**35**

^**a**^Some individuals have multiple skill areas, so total numbers in this table are greater than the number of individuals (n = 44) who advised on development of the App

**Fig. 1:**
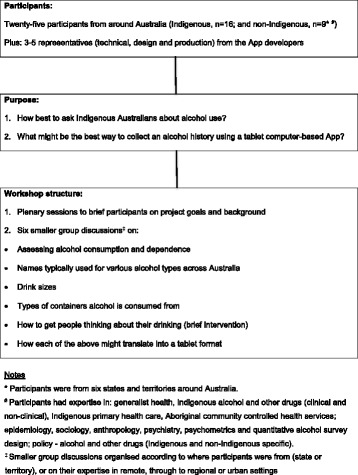
Purpose and structure of a two-day workshop to seek expert advice on development of the Grog Survey App

The main steps taken to develop the App are described below:

- Review of the design of key selected national and international alcohol surveys using peer-reviewed and grey literature to compile a broad list of potential survey items
- Review of relevant websites and Apps to compile a list of potential technical features
- An external company (“We are the Nest/Frost Collective”) awarded the tender to develop and build the App
- Survey items drafted and comment sought from investigators and other colleagues
- Two-day consultation workshop for 25 participants from around Australia (Indigenous, *n* = 16; and non-Indigenous, *n* = 9; see Fig. 1)
- Survey items finalised using feedback from the workshop, from investigators and relevant colleagues. Questions selected on demographics, alcohol consumption (see Table 2), dependence, harms to self or others, treatment access and collecting feedback about the experience of using the App
- Development of the App and user testing: Workshop participants, investigators and colleagues tested the App, with comments reviewed by two authors (Lee and Conigrave) then submitted to the developers

**Table 2:** Comparisons between a selection of consumption items on the Australian National Drug Strategy Household Survey, AUDIT and the Grog^a^ Survey App

Existing survey item	Response categories	Wording changes	Response categories	Technical solutions	Survey delivery solutions
Have you had an alcoholic drink of any kind in the last 12 months?^b^ [NDSHS]	Yes, No	Have you had any grog^a^ at all in the last 12 months? (Since the [Easter holidays] last year)^c^	Yes, No	The App dynamically calculates which reference point to use (for ‘in the last 12-months e.g. Easter holidays versus New Year) and inserts this into the survey question (text on screen and in audio)	Headphones supplied for privacy and anonymity; research assistant sitting a little away if assistance needed
In the last 12 months, how often did you have an alcoholic drink of any kind?^b^[NDSHS]	• Every day • 5 to 6 days a week • 3 to 4 days a week • 1 to 2 days a week • 2 to 3 days a month • About 1 day a month • Less often • No longer drink	Some people drink grog most days while others drink ‘once in a blue moon’^d^. How often do you drink grog at all?^e^	• ‘Once in a blue moon’^d^ • Sometimes • A few times a week • Most days or every day	–	–
How often do you have a drink containing alcohol? [AUDIT Q1]	• Never • Monthly or less • 2-4 times a month • 2-3 times week • 4 or more times a week				
How often do you have six or more standard drinks on one occasion? [AUDIT Q3]	• Never • Less than monthly • Monthly • Weekly • Daily or almost daily	Thinking of the last 12-months [since Easter last year], how often would you drink [this much grog or more] in 1 day (24 h)?^e^	• Never • ‘Once in a blue moon’^d^(less than once a month) • Sometimes (1-3 times a month) • A few times a week (1-3 times a week) • Most days or every day	Interactive Dynamically shows image equivalent of 5 standard drinks (50 g) of the alcohol type most often consumed based on the last 4 drinking occasions.	–

^a^Slang for ‘alcohol’

^b^From the 2013 Australian National Drug Strategy Household Survey

^c^Example of reference points used to anchor answers reflecting on ‘in the last 12-months’ time period

^d^Slang for ‘rarely’

^e^Example of using a conversational way to ask about drinking, often with a gentle introduction to the question

## Results

Here results of consultations are summarized and the general principles and key features underpinning the survey items and their delivery in the App are described.

### General principles

Consultation suggested that the App would need to:

1. Be suitable for an Indigenous Australians aged from 16 to old-age, including those who are unfamiliar with computers or tablets;
2. Be suitable for individuals in urban through to isolated or traditional areas;
3. Help individuals to be comfortable telling their drinking story (e.g. how often or how much);
4. Reassure participants of confidentiality;
5. Work offline, then data from each iPad can be ‘pushed’ to a secured computer server at the University of Sydney when WIFI is available;
6. Provide a de-identified summary of completed surveys periodically (sex, age, community, drinking status), with remote access to data for principal investigators; and
7. Be comparable with (some items of) existing national and international alcohol surveys or screening tools.

### Suitability of existing approaches to measure alcohol consumption

Examples of national and international alcohol surveys were reviewed, and potential items that might be adapted for use on the App were discussed with Indigenous and non-Indigenous experts. International tools included: Alcohol Use Disorders Identification Test (AUDIT) [15]; Composite International Diagnostic Interview Version 7.1 (CIDI) [16]; Alcohol, Smoking and Substance Involvement Screening Test Version 3.0 (ASSIST) [17]; 2007 Gender Alcohol and Culture: An International Study Survey Version 6.1 (GENACIS) [18]; and the International Alcohol Control Policy Evaluation Study (IACS) [19]. Australian tools included: the 2008 National Aboriginal and Torres Strait Islander Social Survey (NATSISS) [20]; the 2013 National Drug Strategy Household Survey [21]; the Indigenous Risk Impact Screen (IRIS) [22]; the Harms From Others’ Drinking Study [23]; and a community survey on alcohol consumption in Indigenous populations in remote Western Australia [24].

Two other internationally validated approaches to assessing alcohol use were considered, as they appeared to have particular relevance to Indigenous Australian contexts. The ‘Timeline follow back’ approach [25] encourages the individual to recall where they were and who they were with, to help elucidate a detailed history of drinking. The interviewer works backwards, day-by-day for the past month or up to a year. This recounting of real life context and linking of drinking to events and people was considered relevant and approachable in an Indigenous context. However, substantial time is needed to use this approach in its entirety. Another approach, the “Finnish” method, only enquires into the last four drinking occasions [26] and so is less time consuming. Again, the Finnish method asks the person to think over the events or context that were associated with drinking, and does not assume regularity of drinking pattern. However, it is possible that the past four drinking occasions may not be typical of the rest of the year: for example, a person might have 4 days of heavy drinking associated with a trip to the city, while the rest of the year was spent in a ‘dry’ (alcohol-restricted) area.

Item selection was then guided by Indigenous and non-Indigenous colleagues’ advice, for example that:

1. It should take no more than 20 min to complete the survey App (due to competing demands on participants);
2. The app should make it easier for individuals to describe their drinking without requiring skills in numeracy or literacy; and
3. Survey items should cater to a range of drinker types (e.g. including those who drink episodically).

For quantifying alcohol consumption: 10 items are enquired into. This consisted of:

1. Any alcohol consumption in the last year;
2. Frequency of consumption in the last year (The Alcohol Use Disorders Identification Test [AUDIT] Q1-modified);
3. The frequency and timing of the last four occasions of drinking and what was consumed, using the ‘Finnish method’ [25], combined with elements of ‘time line follow back’ [24] to help participants remember where they were and who they were with in each drinking occasion;
4. Reasons why participants sometimes might drink more;
5. Quantity and types of alcohol consumed in a heavy drinking occasion (24-h period);
6. Length of the longest period of no drinking in the last year; and.
7. Frequency of consuming five or more standard drinks (24 h period) in the last year? (AUDIT Q3-modified) with visual cues to quantify.

We were advised that AUDIT-Q2 was problematic as it asks into ‘usual’ consumption, and in some traditional regions, the concept of ‘usual’ does not exist. The app also collected other data on alcohol use behaviours that are the not the focus of this paper (on: alcohol dependence, harms to self or others and treatment access).

### Presenting questions in a conversational way

Indigenous colleagues and other clinicians stressed the importance of asking questions in a conversational manner. This was based on clinical and research experience, and on the work of one author (Assan and colleagues) on training clinicians in the use of the Indigenous Risk Impact Screen (IRIS; a screening tool for alcohol, drug and mental health issues developed by and for Indigenous Australians [22]). Accordingly, sensitive survey items were introduced with a short scenario (where appropriate), to assist the individual to reflect on their own life experience (see Table 2).

To ensure privacy, each participant would be presented with an iPad and headphones and be supported by an Indigenous research assistant to open the survey. The individuals would then work through the questions, with a research assistant sitting a little distance away in case problems or questions arose.

### Reference points used to ‘anchor’ time periods

Time is not universally understood as a linear concept in Australia [10, 11]. In traditional communities time of year may be marked more by the seasons or a tree flowering, or times when shops are shut rather than by a calendar. So reference points were used to help individuals to anchor their answers in time. Based on a small group discussion focused on this issue at the workshop, reference points that are widely recognised across Indigenous Australia were agreed upon. As a result, the ‘last 12-months’ is divided into quarters with the help of four key time points: a) Christmas or New Year (December/January); b) Easter (April); c) National Aboriginal and Islander Day of Celebration (NAIDOC) week (July); and d) Australian Football League (AFL) or National Rugby League (NRL) grand finals (September/October; see Table 2). There was consensus that individuals who do not celebrate Christmas or Easter, or who do not follow sport, would know when in a calendar year these events occur.

The survey app calculates which reference point (for ‘in the last 12-months’) to use depending on the date when the App is being completed. This then enables an individual to focus on what they were doing, for example, at ‘Easter last year’, rather than trying to remember what they were doing ‘12-months ago’. A visual timeline was used to allow respondents to select dates moving back in time, of their four recent drinking occasions. The reference points are converted to dates ‘behind’ the App for data analysis.

### Response categories for questions on frequency of drinking

Indigenous colleagues and other clinicians advised that response categories typically used in alcohol surveys posed difficulties, as they are reliant on individuals counting days, weeks or months [10]. Instead, modified response categories were used that included colloquial English that would be commonly understood by the target population (e.g. ‘once in a blue moon (less than once a month)’ instead of ‘less than monthly’; see Table 2).

### Asking about pattern and quantity of drinking on the iPad App

The last four occasions approach [26] was adapted for a user-friendly and visual approach. This combined elements of ‘Timeline follow back’ [25], and was seen by our advisers as compatible with a conversational or story telling approach.

On the iPad screen, a retrospective “grog diary” appears as a strip. The participant selects when (in the last 12-months) each of their last four drinking occasions occurred. The time periods displayed on the first screen are: “Yesterday, 2 days ago […. up to], 1 week ago etc”. The user moves backwards in time to select the day. The App uses the timing of drinking and the quantity selected (see below) to calculate average quantity consumed.

In addition, to better describe drinking which may stop and start according to geographic location or circumstance, participants are asked about their longest gap without alcohol in the last 12-months (indicating the actual length of time using the same retrospective grog diary and nominating reasons for this dry period).

Each individual is also asked about a ‘heavy’ drinking occasion: “In the last 12 months, when you drank a lot of grog, would you ever drink more than [this--]?”. An image depicting the largest amount of alcohol that the person reported consuming in the last four drinking occasions is then shown. If the person responds that they sometimes drink more than that amount, they can select items of alcohol to describe their level of consumption at that higher level of drinking. The individual then reports how *often* they drink a lot for them (see ‘Response categories for questions on frequency of drinking’ above).

### Identifying the type of alcohol a person drinks

Some drinkers are not familiar with the names for some categories of alcohol type, for example, ‘fortified wines’, but rather they identify type of alcohol by its brand or container. To address this issue, a simplified classification of alcohol types was agreed on: beer, wine, port or sherry, spirits or other. Pictures of common local brands in each alcohol type would be displayed. The ‘other’ category included cocktails, methylated spirits and drinks not listed elsewhere.

A listing of common alcohol brands and drinking containers was created for each alcohol type in each surveyed state. With the help of colleagues from those states, this guide was refined to reflect popular alcohol brands but also sufficient choice in each alcohol type. State-based drinking preferences were reflected. For example, “XXXX” was a beer choice made available for Queensland individuals, but instead, “West End” appeared in SA. It was not possible to present every choice, so research assistants were instructed to encourage participants who cannot find their choice to select an alcohol type of similar strength to their preferred brand, or to choose the “other” category.

Beer posed particular challenges as Indigenous colleagues and other clinicians reported confusion around terms such as ‘regular strength’ versus ‘mid strength’ or ‘low alcohol’ [21]. The term ‘low carb’ was sometimes incorrectly understood to mean ‘low alcohol’. To reduce confusion, pictures of several actual beer brands were used. Brand recognition is typically strong. For example, workshop participants advised that in more isolated settings, some types of alcohol are known by the colour of the packaging. So, if hand drawn images were to be used, extra care would need to be taken to ensure comprehension of brand names in different geographical settings.

### Drink containers

**Fig. 2:**
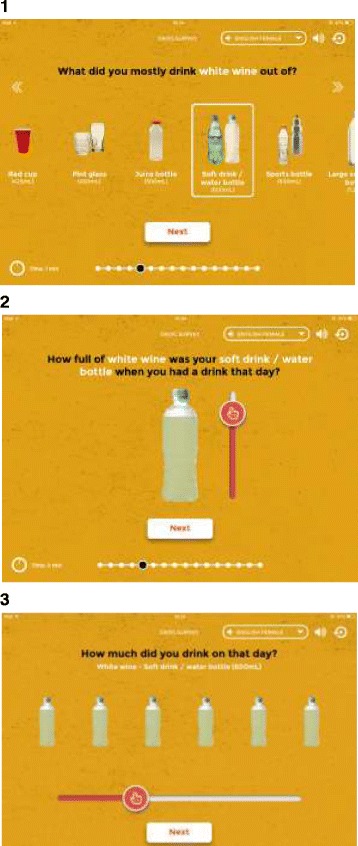
Self-reporting alcohol consumption using the Grog Survey App

Indigenous colleagues stressed the importance of offering a broad range of containers from which alcohol might be consumed. For example, many individuals do not drink wine from a wine glass, especially in remote communities. Instead they may use a container sold for other purposes, ranging from a pannikin (metal mug; 355 mL), slurpee/slushy cup (490 mL), empty water bottle (600 mL), through to a large soft drink bottle (1.25 L; see Fig. 2).

### Working out individual consumption based on a share of what the whole group drank

**Fig. 3:**
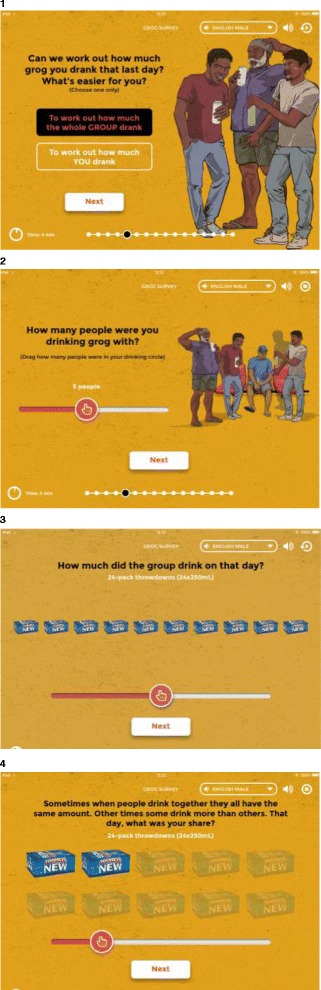
Self-reporting alcohol consumption from what the whole group drank using the Grog Survey App

Indigenous and non-Indigenous clinicians reported that when collecting an alcohol history, some clients spontaneously report what the whole group had to drink, rather than on what they alone consumed. The clinician then assists the individual to estimate their share. So, when asked about the last drinking occasion, the App enables the participant to choose to describe what they consumed as an individual or to describe what the group drank (see Fig. 3).

After selecting when in the last 12-months the last drinking occasion took place (on the retrospective grog diary), an individual is asked: “How many people were you drinking with?”. A ‘slider’ (from left to right) enables the individual to select: “Just you” through to a group of “10+ people”. The accompanying image changes as the slider is moved to show the number of people in the drinking group. The individual then chooses 1) To work out how much the whole group drank or 2) To work out how much they themselves drank.

Then, for example, if an individual selects that the last time they drank, the group consumed ten cases or slabs (24-cans) of full strength beer. The App then asks: “Sometimes when people drink together they all have the same amount. Other times some drink more than others. That day, what was your share?” In the screenshot below (Fig. 3), an individual reported a total of five people in their drinking group. So, the next screen defaults to showing an image of two out of ten cases as that individual’s share (i.e. the App assumes an equal share of beer on this occasion). The individual can then slide a meter to adjust their individual portion. Invisible to the user, each container is divided into 10 to calculate standard drinks. For example, of the ten cases of full strength beer consumed by the group, the individual consumed two cases (see Fig. 3). Estimated at 4.8% alcohol/volume at 0.789 g/mL, this equates to 45.5 standard drinks consumed by that individual on that occasion.

### Modifying AUDIT Q3

This item was modified to be in keeping with Australian drinking guidelines (i.e. to ask about consuming *five* drinks or more on an occasion) [7]. Also, instead of relying on participants to convert what they drink into ‘standard drinks’, the App dynamically produces an image of 50 g or more of ethanol based on the type of alcohol that each individual consumed the most of (i.e. the maximum grams of alcohol) from their last four drinking occasions (see Table 2).

### Image and audio options

When a survey is started, each participant identifies their gender. The images, audio and lifestyle references are then matched with that gender. For example, female participants predominantly see women and girls in the images on screen and the audio is spoken by a woman. Original artwork featured on the app was commissioned from an artist employed by the Aboriginal Drug and Alcohol Council SA.

Two language options are offered in the Stage 1 version of the App – English or Pitjantjatjara, a language of Indigenous Australians used in a region of South Australia, Northern Territory and Western Australia. To begin with, two experienced Indigenous alcohol and other drug professionals recorded the English female and then the English male audio. During recording, there was further refinement of the wording of survey items, for example, to check that phrasing was comfortable. Suggestions made by these clinicians during the recording process were checked at the time with three researchers (Lee, Conigrave and Perry).

The Pitjantjatjara language program at the University of South Australia facilitated translation of survey items from English to Pitjantjatjara (including ‘back translation’ of key items). Audio was provided by two Pitjantjatjara language speakers/interpreters (a male and a female). This team and a researcher (Perry) met face-to-face to workshop the translation of survey items. Where there was differing opinion, clarification was sought on intended meaning from a researcher (Perry or Conigrave), until consensus was reached. Efforts were made to ensure suitability of survey items for Pitjantjatjara speakers from adolescence to old age. Further workshopping of survey items occurred in the recording studio. To ensure consistency, one researcher (Perry, who speaks some Pitjantjatjara) was present for one of the English and both of the Pitjantjatjara (male and female) recordings. Suggestions made during the recording process were checked at the time by two researchers (Lee and Perry).

## Discussion

This study describes for the first time the process taken to develop a tablet-based survey ‘App’ to help Indigenous Australians to describe and measure their drinking. The approach described is consistent with earlier interactive touch screen-based platforms that screen for alcohol use (and other risk factors) in Indigenous Australians [27, 28]. However, it extends this work in several ways. While previous studies have focused on screening [27, 28] or health promotion [28], this study sets out to create an alternative way to measure self-reported alcohol consumption in a detailed manner that is similar to approaches used in a household survey. This potentially will provide a gold standard against which shorter screening tools can be validated. Validation of the survey App itself, comparing it with a clinical assessment and also test-retest, is currently being conducted as well as an assessment of its acceptability. These will be reported separately.

Worldwide there are challenges in recording an accurate assessment of drinking [9]. This challenge is greater if alcohol is shared, for example, as is described in Africa and in Indigenous Australia [29–31]. Reliance on individuals to convert their consumption to standard drinks (or units) is fraught. Even in higher socio-economic status populations, comfort with mathematics varies. In lower socio-economic status groups, or in subpopulations with lower literacy in the mainstream language or numeracy, the challenge is greater. This situation is likely to occur increasingly in multicultural societies and with rising numbers of displaced persons.

With any survey platform, getting participants to accurately reflect on their drinking presents a challenge [32]. A survey-based App can harness available technologies to dynamically customize the survey experience for each participant. For example, a conversational style of questions in plain English text can be augmented by audio in the local language with pictures customized to gender and community setting, to help create a relaxed friendly and responsive ‘interview’ environment [33].

Mathematical formulae embedded in the programming back-end can convert the library of images showing actual alcohol products and range of containers in the front-end into standard drinks (or grams of alcohol). This can help the individual to recall what they were drinking without the need to use mental arithmetic to convert drinks consumed into “standard” drinks.

In busy Indigenous primary care settings, some Indigenous health professionals have been reticent to conduct alcohol screening because they may be required to screen their own family members or friends [34]. Also, individuals may be reticent to take part in alcohol screening (or surveys) because of past experience of racism and discrimination [35]. The App enables participants to ‘anonymously’ tell their alcohol story without needing to make personal disclosures to a research assistant or health professional. Even if the research assistant knows the individual, individuals are informed during recruitment and explanation of the study that data cannot be accessed or linked to an individual once entered into the App.

This appealing format of the App is also likely to result in higher response rates. In field-testing to date, research assistants report being inundated by community members wanting to try out the App having heard about it via ‘word of mouth’ (personal communication with J Perry, S Wilson, N Hayman; qualitative feedback from staff will be reported in a later paper).

### Limitations

In this work, an iterative process of advice seeking was used to develop the App instead of formal focus groups or semi-structured interviews. However, such an approach allowed us to work from the ‘ground up’, collaborating with the broadest range of colleagues and to reflect varied viewpoints [6, 36].

The number of survey items that could be included was limited, as it was recommended that the duration of the App survey should be no more than 20 min. This suited the target population who are often time-poor and where there can be many distractions (such as the needs of children or relatives). It also suited the Indigenous primary care services and other drug and alcohol facilities where recruitment occurred, where time pressure is reported as a significant barrier to alcohol screening [34, 37].

### Validation of the App

Between August 2016 to May 2017, a pilot version of the survey is being administered to Aboriginal or Torres Strait Islander respondents in three rural and remote sites in South Australia and one urban site in Queensland. The Queensland site was likely to recruit Aboriginal or Torres Strait Islander respondents, given its proximity to the Torres Strait and Papua New Guinea. The Aboriginal field research assistants who administered the survey in Queensland are known to the community and are aware of particular issues in relation to alcohol and other drug use in individuals from a Torres Strait background [10]. The responses to the app will be compared with a clinical assessment conducted by an Indigenous health professional, and with a repeat administration of the survey App (2-7 days later). Analysis will examine the internal and external validity of the app, test-re-test reliability, acceptability and feasibility. The App converts the amount and strength of alcohol consumed into the equivalent number of Australian standard drinks (each 10 g ethanol). The consumption on each of the last four drinking sessions, and the number of days between sessions are used to estimate the average number of standard drinks per drinking day, the number of drinking days per year and total volume consumed for the year in grams of ethanol (and then in Australian standard drinks). In addition, consumption on the heaviest drinking session is calculated. Efforts will then be made to further shorten the duration of the App survey.

### Future applications

Household surveys: The App could be a cost-effective way to collect and store confidential survey data, even on a national level. It can operate ‘off line’ so is suitable for isolated settings where there may be little or no internet access. There would be an initial outlay to conduct comprehensive translations and back translations [38] and audio recordings in different languages. However, such recordings are cheaper than having translators present during each administration of the survey. Also, audio recording ensures standardization of instructions across all surveys.

Research in general populations: The technology used to create the survey App could have broader benefits beyond this field of research. Instead of needing to store confidential paper surveys while out in the field, data are simply synchronized daily from each tablet-computer to a secure central point (e.g. university or government server). This can be performed using wireless internet tethered to a smart phone or other wireless modem. Study leaders can also access data remotely during data collection. This allows for monitoring of study progress in ‘real’ time (e.g. to check quotas of data collected, better support research assistants in the field).

Computer screening in clinic waiting rooms: The App could be modified to improve the way alcohol screening and assessment is conducted with clients. For example, clients could be handed a tablet (and headphones) in the waiting room and asked to complete a survey. Completed surveys could then be shared with the treating health professional with the client’s consent. This could help the health professional to become aware of possible unhealthy drinking [39] including any risk of alcohol withdrawal. Further work is needed to better understand the effectiveness of brief intervention among Indigenous populations [40]. However, more accurate assessment of drinking will improve both screening and outcome measurement.

The guidance into ways of asking an alcohol history, obtained from consultation for this app, is very relevant to clinicians. This includes making it easier for patients to relate to time points marking the last 12-months. Also, clinicians can avoid making patients do mental arithmetic, in either self-reporting their drinking or when the clinician is conveying drinking guidelines.

Health promotion role: National drinking guidelines are often expressed in standard drinks or units, which can be hard for the individual to relate to their own drinking. The Grog Survey App could help a person to first quantify their drinking, and then to compare this against current drinking guidelines.

## Conclusion

Estimating alcohol consumption is challenging in any setting [41]. It is made more so when alcohol is shared or there may be only intermittent access to alcohol. This requires a ‘shake up’ of existing ways of asking about alcohol consumption in surveys or clinical practice. Interactive tablet-based technologies potentially enable some of these challenges to be overcome. The detailed and iterative advice provided by a range of content experts helped to create a survey App that was respectful of a range of viewpoints (cultural, clinical, health promotion, policy, research etc). The approach taken to develop the App and its key features are likely to be useful for a wide range of marginalized populations. They are also relevant to assessing drinking in the developing world, where drinking is often in unstandardized containers. Among vulnerable groups the need for an accurate estimation of alcohol consumption is particularly important to inform prevention and treatment efforts.
