# Correction to: Developing a tablet computer-based application (‘App’) to measure self-reported alcohol consumption in Indigenous Australians

**DOI:** 10.1186/s12911-018-0604-z

**Published:** 2018-05-02

**Authors:** K. S. Kylie Lee, Scott Wilson, Jimmy Perry, Robin Room, Sarah Callinan, Robert Assan, Noel Hayman, Tanya Chikritzhs, Dennis Gray, Edward Wilkes, Peter Jack, Katherine M. Conigrave

**Affiliations:** 1 University of Sydney, Discipline of Addiction Medicine, Indigenous Health and Substance Use, NHMRC Centre of Research Excellence in Indigenous Health and Alcohol, King George V Building, 83-117 Missenden Road, Camperdown, NSW 2050 Australia; 2 Centre for Alcohol Policy Research, LaTrobe University, 215 Franklin Street, Melbourne, VIC 3000 Australia; 3 Aboriginal Drug and Alcohol Council (ADAC) South Australia, 155 Holbrooks Road Underdale, Adelaide, South Australia 5032 Australia; 4 Alcohol, Tobacco and other Drugs Service, Queensland Health, 190 Palmerston Vincent, Townsville, QLD 4814 Australia; 5 Southern Queensland Centre of Excellence in Aboriginal and Torres Strait Islander Primary Health Care, 37 Wirraway Parade, Inala, QLD 4077 Australia; 6 School of Medicine, University of Queensland, Herston Road, Brisbane, QLD 4006 Australia; 7 School of Medicine, Griffith University, Gold Coast Campus, Gold Coast, Brisbane, QLD 4222 Australia; 8 National Drug Research Institute, Curtin University, 10 Selby St, Shenton Park, WA 6008 Australia; 9 Drug Health Services, Royal Prince Alfred Hospital, Sydney Local Health District, KGV Building, Missenden Road, Camperdown, NSW 2050 Australia

## Correction

After publication of the original article [1] it was noted that the name of author, Peter Jack, was erroneously typeset in both the PDF and online formats of the manuscript as Peter Jack GradDipIndigH.

This inclusion of his qualification as part of his surname was introduced during typesetting, thus the publisher apologises for this error. The original article has also been updated to reflect this correction.
